# A Study on Mechanical Properties of Low-Cost Thermoplastic-Based Materials for Material Extrusion Additive Manufacturing

**DOI:** 10.3390/polym15142981

**Published:** 2023-07-08

**Authors:** Mihaela-Raluca Condruz, Alexandru Paraschiv, Teodor-Adrian Badea, Daniel Useriu, Tiberius-Florian Frigioescu, Gabriel Badea, Grigore Cican

**Affiliations:** Romanian Research and Development Institute for Gas Turbines COMOTI, 220D Iuliu Maniu Av., 061126 Bucharest, Romania; raluca.condruz@comoti.ro (M.-R.C.); teodor.badea@comoti.ro (T.-A.B.); daniel.useriu@comoti.ro (D.U.); tiberius.frigioescu@comoti.ro (T.-F.F.); gabriel.badea@comoti.ro (G.B.); grigore.cican@comoti.ro (G.C.)

**Keywords:** extrusion, additive manufacturing, tensile, open-hole tensile, composites

## Abstract

The present research focused on studying the mechanical properties of three commercially available thermoplastic-based materials used for the additive manufacturing (AM) fused filament deposition (FFD) method. The scientific motivation for the study was the limited information available in the literature regarding the materials’ properties, the inconsistencies that were recorded by other scientists between the materials’ properties and the technical datasheets and the anisotropic behavior of additively manufactured materials. Thereby, it was considered of great importance to perform an extensive study on several materials’ mechanical properties, such as tensile properties and flexural properties. Three materials were tested, Tough PLA, nGen CF10 and UltraFuse PAHT CF15. The tests consisted of monotonic tensile tests, open-hole tensile tests and three-point bending tests. The tests were assisted also with the use of microscopical investigations. Framed specimens’ configurations with two different raster orientations (90°/0° and −45°/+45°) were manufactured using an in-house-developed 3D printing equipment. The best mechanical performances were recorded for UltraFuse PAHT CF15. The 90°/0° raster orientations ensured the highest tensile, open-hole tensile and flexural strength, regardless of the material type, while the −45°/+45° raster orientations ensured the highest elongation values. The analysis showed the importance of the experimental validation of materials for AM.

## 1. Introduction

In recent times, AM has garnered considerable attention, as it can produce complex shaped parts for various applications. According to ISO/ASTM 52900 [1], there are seven AM process categories or methods based on the functioning principle of AM equipment and materials used as feedstock. Since the development of AM, extensive research has been conducted in the field on all types of processes and materials (metals [2], ceramic materials [3], thermoplastics [4,5,6] and composites [7]).

Currently, metallic and ceramic AM includes very expensive processes and materials, the most inexpensive AM process being material extrusion (MEX), where the material is selectively dispensed through a nozzle [1]. MEX is usually used for materials bonded using chemical reactions or thermal reactions [1], meaning mainly thermoplastic materials, and it is the most used for research purposes and home applications. A specific MEX process is fused filament deposition (FFD) or fused filament fabrication (FFF), which deposits layer-by-layer thermoplastic materials and thermoplastic matrix composites according to a 3D CAD model.

Many desktop FFF machines are currently available, including at low prices, and are used to manufacture parts that do not require excessive mechanical strength. Various studies have been conducted over the years on FFF-manufactured parts, including studies on design for FFF [8], machine components and functionality [9], mechanical properties of FFF-manufactured parts [10] and the mechanical properties of materials for FFF [11,12].

For example, Salem et al. [13] studied the tensile properties of PLA (polylactic acid) in raw conditions and after 3D printing, because the properties given by the material supplier are not the same as the properties of the final part. The findings revealed a significant disparity between the performance of printed materials and the specifications outlined in their datasheets. Their conclusion highlighted the need to perform a testing campaign for materials designed for AM to select the proper material for the application. Grabowik et al. [14] studied the tensile properties of different filaments: 3DGO Wood (a PLA reinforced with wooden powder), PLA, ASA (acrylonitrile styrene acrylate), PET (poly(ethylene terephthalate)), ABS (acrylonitrile butadiene styrene) and PMMA (poly(methyl methacrylate)). The datasheets included information regarding the properties of the materials in one printing direction, thereby the actual printing direction was selected based on the information available in the datasheet to ensure the materials’ best mechanical performances. They concluded that the lowest performances were recorded for 3DGO Wood and the best performances were recorded for PMMA, even if PET was easier to print and could be considered a good material for tough applications.

Farbman et al. [15] investigated the mechanical properties of ABS and PLA using experimental and computational methods. They varied the material, infill percentage, geometry, load orientation and strain rate, concluding that the materials’ properties depended on various factors and special attention needed to be paid to obtain high-quality printed specimens. Morettini et al. [16] also studied the mechanical and physical properties of PLA parts manufactured through FFF. An average ultimate tensile strength (UTS) of 57.15 MPa and an elastic modulus of 2606 MPa were obtained for horizontally printed specimens. Catana et al. [17] published a study related to the flexural properties of PLA and PLA reinforced with short glass fibers bars and tubes with different cross sections (circular and ellipse) determined with three-point bending tests. The bending strength of approximately 64 MPa for unreinforced PLA (tube configuration) and 82 MPa for hybrid specimens consisted of an outer arrangement of 31% and an inner arrangement of 69% PLA–glass.

The mechanical properties of additively manufactured PLA and ABS structures (AMed) were investigated by Ozsoy et al. [18] using tensile, compression and three-point bending tests. The results showed that the mechanical properties of the materials depended on the filling density, and could be optimized by increasing the filling density rate, thereby decreasing the printing speed. For PLA, the following values were obtained: tensile yield strength of 37.33 MPa, 1.080 GPa elastic modulus, 32.45 MPa compression yield strength, 0.834 GPa elastic modulus, 37.39 MPa flexural yield strength and 1.547 GPa elastic modulus; lower properties were recorded for ABS. Sodeifian et al. [19] studied the mechanical properties of pure polypropylene (PP)/glass fiber (GF) and PP/GF composites containing maleic anhydride polyolefin (POE-g-MA) at different weight percentages (10, 20 and 30 wt.%). Based on experimental tests, it was observed that the addition of glass fiber enhanced the strength and modulus while reducing the flexibility of the material.

Sang et al. [20] researched the development of short-basalt-fiber/short-carbon-fiber-reinforced PLA (PLA/KBF and PLA/CF) composites for 3D printing applications. They observed that PLA/KBF showed comparable tensile properties and higher flexural properties than PLA/CF, which could be explained by the high complex viscosity of PLA/CF affecting the interlayer adhesion. For neat PLA, a 54.2 MPa tensile strength was recorded, while a 71.9 MPa maximum tensile strength was recorded for 20% reinforced PLA/KBF. For PLA/CF, the maximum tensile strength was recorded for the specimens with 10% CF (69 MPa), while increasing the CF weight fraction from 10% to 20% ensured a decrease in mechanical properties. Ning et al. [21] investigated the properties of customized carbon-fiber-reinforced ABS for 3D printing. They manufactured FDM (fused deposition molding) filaments using virgin ABS pellets and carbon fiber powders. Different reinforcement percentages were used (3 wt.%, 5 wt.%, 7.5 wt.%, 10 wt.% and 15 wt.%), and it was concluded that the addition of carbon fibers could enhance the tensile strength and elastic modulus. However, it could lead to a reduction in toughness, yield strength and ductility.

Ahmadifar et al. [22] studied the mechanical behavior of polymer-based composites obtained through the use of FFF. They used raw materials from Markforged, namely, Onyx (a reinforced polyamide PA6 with 6.5 wt.% chopped carbon fibers) and CF-PA6 additionally reinforced with continuous glass fibers (selectively added only on some layers). They used many infill patterns and different printing directions for the reinforcement layers, and obtained a homogenous distribution of carbon fibers in Onyx; the CF-PA6 specimens with solid infill patterns exhibited remarkable stiffness and mechanical properties under tension (tensile strength of 30.31 MPa).

Tutar [23] conducted a comparative evaluation of the process parameters on the mechanical properties of AMed PA (UltraFuse PA) and CF-PA (UltraFuse PAHT CF15) materials. He performed tests on framed and unframed specimens with various infill raster orientations (0°, 45°, ±45° and 90°). The tensile strength of the PA specimens was in the range of 24.6–40.5 MPa, while for the unframed PA-CF, it was between 31.4 and 95.4 MPa, and for framed PA-CF, it was between 88 and 106.6 MPa, depending on the raster angle. In his conclusion, it was observed that carbon fiber reinforcement, irrespective of the raster angle, resulted in a reduced toughness and ductility while at the same time enhancing the tensile strength and stiffness.

Open-hole tensile testing is a representative testing procedure for fastened structures, where the hole is a stress concentrator [24]. These tests are an effective approach to predict how a fixed part can behave under various loads [25,26]. Open-hole tests were reported in the case of composites subjected to tensile or compressive loads [27,28]. They are significant for industrial applications where composite materials are used, for example, in the aerospace and automotive industries [24].

The FFF process is efficient and low-cost, and can be used to manufacture various parts, including structural components for unmanned aerial vehicles (UAVs). Several studies have been published on additive-manufactured UAVs or their components [29,30,31,32,33,34,35].

As highlighted by other authors, the technical datasheets of materials for additive manufacturing give the materials’ characteristics as obtained in specific conditions or are incomplete; therefore, before designing and manufacturing a part, the materials’ properties should be experimentally determined.

This study aimed to evaluate the mechanical properties of three different low-cost materials for FFF intended for the structural part manufacturing of an experimental UAV.

## 2. Materials and Methods

The mechanical properties of three different materials for FFF were assessed based on tensile, open-hole tensile and three-point-bending tests. The materials selected for the research were commercially available and consisted of filaments of *Tough PLA black* (polylactic acid) produced by FormFutura (Amsterdam, The Netherlands), *nGen CF10 black* (PETG—polyethylene terephthalate glycol—created with Amphora AM3300 polymeric material filled with 10 wt.% short carbon fibers) produced by colorFabb (Belfeld, The Netherlands) and *UltraFuse PAHT CF15 black* (a high-temperature polyamide-based filament filled with 15 wt.% short carbon fibers produced by BASF-Innofil3D, Emmen, The Netherlands). The materials’ properties according to their datasheets are presented in Table 1.

Specimens were manufactured using an in-house-developed 3D printer that could ensure a printable area of 300 × 300 × 350 mm (L × l × h). For all manufacturing processes, a tungsten nozzle of 0.6 mm in diameter was selected to ensure homogeneity between specimen geometry tolerances and layer configurations. Moreover, the nozzle diameter was selected based on the minimum diameter of the filament used to prevent the clogging of the nozzle.

The specimens’ 3D CAD models were realized using SolidEdge 2021 software (Siemens, Munich, Germany). The specimens’ dimensions and testing conditions were established based on active ASTM/ISO standards applied for polymeric composites, as shown in Table 2.

**Figure 1:**
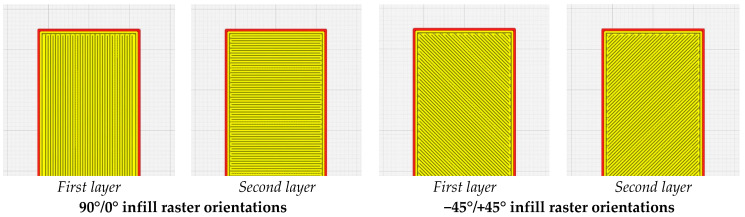
Representative images with the first and second layers of specimens; images from Cura software.

The STL models were processed (slicing) using Cura 5.3.0 software (UltiMaker, a free processing software, Utrecht, the Netherlands). To prevent the clogging of the nozzle and to lower the time required to manufacture a specimen, a height of 0.36 mm was used in slicing the model with a first layer of 0.2 mm in height. The values selected were in the recommended range of 25–75% of the diameter of the nozzle. The resulting G-code was inserted into the machine computer. All specimens were designed as framed configurations (2-frame layers) with full infills and two different raster orientations, 90°/0° and −45°/+45°, as can be observed in the images in Figure 1.

**Figure 2:**
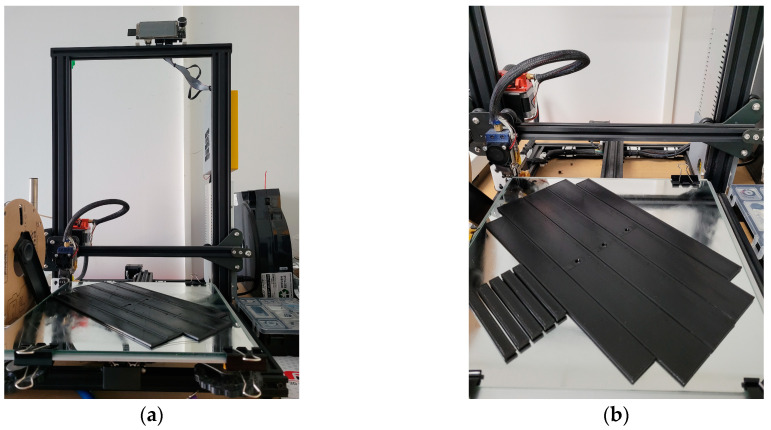
Images during the specimens’ manufacturing process: (**a**) overall image with the 3D printer; (**b**) specimen during the manufacturing process.

The building plate consisted of clear glass covered with Magigoo bed prep adhesive (Thought3D Ltd., Qormi, Malta) to ensure adhesion during the entire printing process. Moreover, before starting each manufacturing cycle, the filament rolls were dried in an oven at 75 °C for 7 h. The manufacturing conditions were established according to the materials’ datasheets and are presented in Table 3. Images from the manufacturing process can be observed in Figure 2.

All mechanical tests were executed in normal ambient conditions using an Instron 3369 testing machine (Instron, Norwood, MA, USA) equipped with a load cell of 50 kN. The testing direction of the tensile and open-hole tensile tests was in the same direction, the 90° raster orientation. After testing, the failure modes were determined for the tensile and open-hole tensile specimens according to ASTM D 3039/D3039M [40] and ASTM D5766/D5766M [41] codifications. The failure modes were established through a visual analysis.

A fracture surface analysis was realized using an FEI Inspect F50 scanning electron microscope—SEM (FEI Company, Brno, Czech Republic). After performing the mechanical tests, sections from the tensile damaged specimens were cut and sputter-coated with gold using SC7620 Mini Sputter Coater/Glow Discharge System (Quorum Technologies, Laughton, East Sussex, UK) to prepare samples for the SEM analysis.

Optic microstructural investigations were also conducted on samples manufactured from all three materials using the same raster orientations (90°/0° and −45°/+45°) just to emphasize several microstructural characteristics of the specimens. For this analysis, we used the Axio Vert.A1 MAT optical microscope with a camera (Carl Zeiss Microscopy GmbH, Jena, Germany). The samples were metallographically prepared through cutting, grinding on sandpaper and felt-polishing using a 1 µm diamond polishing suspension.

## 3. Results and Discussion

### 3.1. Optic Microstructural Analysis

**Figure 3:**
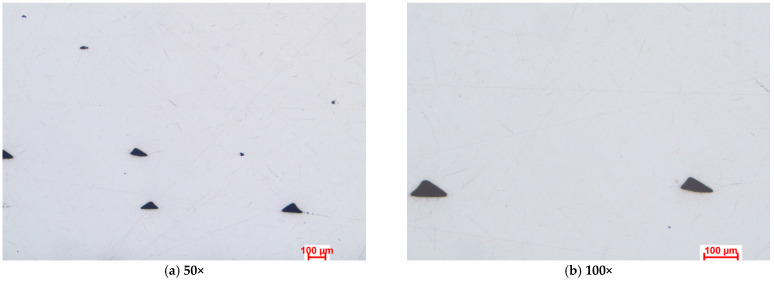

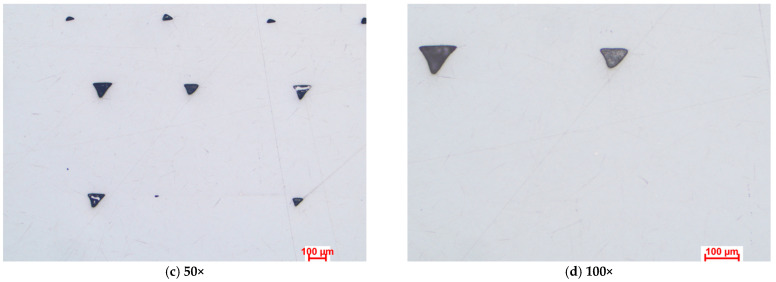
Optical microstructures of samples manufactured with Tough PLA with the two raster orientations (**a**,**b**) −45°/+45° and (**c**,**d**) and 90°/0°.

**Figure 4:**
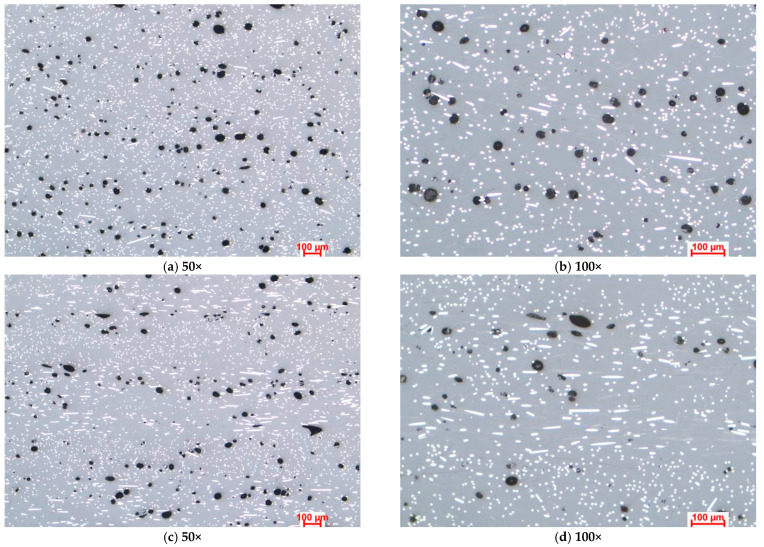
Optical microstructures of samples manufactured with nGen CF 10 with the two raster orientations (**a**,**b**) −45°/+45° and (**c**,**d**) and 90°/0°.

**Figure 5:**
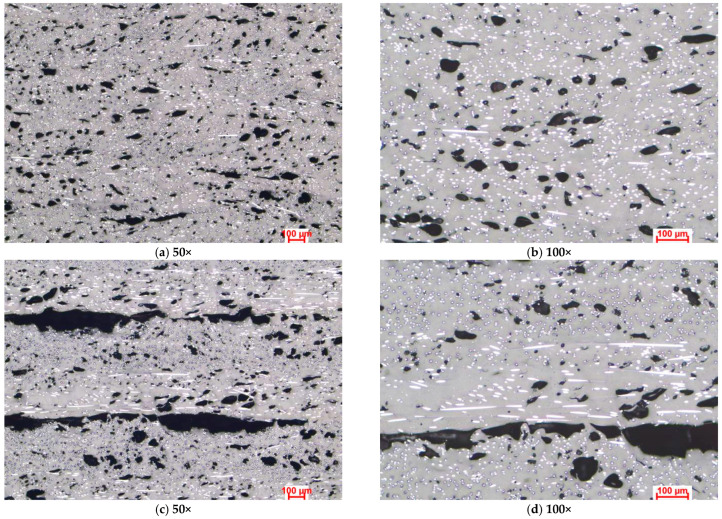
Optical microstructures of samples manufactured with UltraFuse PAHT CF15 with the two raster orientations (**a**,**b**) −45°/+45° and (**c**,**d**) and 90°/0°.

Figure 3, Figure 4 and Figure 5 present the optical microstructures of the samples manufactured with Tough PLA, nGen CF10 (colorFabb, Belfeld, The Netherlands) and UltraFuse PAHT CF15 (BASF, Emmen, The Netherlands) at 50× and 100× magnifications for the two raster orientations of 90°/0° and +45°/−45°, respectively. In these images, pores were visible as black areas with various geometries, while carbon fibers were visible as luminous white spots and lines. The thermoplastic polymeric matrix was grey regardless of the material used.

The micrographs presented in Figure 3, Figure 4 and Figure 5 indicated significant porosities between the overlaid layers in all specimens, irrespective of the raster orientation. At a microscopic level, for the Tough PLA and nGen CF 10 materials, the layer limits were not visible, as observed in the case of UltraFuse PAHT CF15. These pores were a consequence of the stacking effect commonly observed in the FDM process, exhibiting a triangular shape, as highlighted in Figure 3a,b. This effect occurred due to the filament deposition direction alternating, resulting in incomplete filling between the deposition layers. Cracks typically initiate from these pores and propagate, leading to premature material failure. Under mechanical stress, the triangular-shaped pores deformed along with the material, resulting in the formation of discontinuities within the material. These discontinuities could be observed in the optical images and were more pronounced in the case of the UltraFuse PAHT CF15 specimens, as shown in Figure 5a,b. Moreover, smaller irregular pores could be seen within the layers of both the UltraFuse PAHT CF15 and nGen CF 10 specimens, as illustrated in Figure 4 and Figure 5. These materials are hydrophobic and can absorb humidity from the environment. This was evident from the high porosity content observed in the material, even after undergoing a heat treatment in the oven before each manufacturing cycle. The carbon fibers were prominently visible in both configurations of the UltraFuse PAHT CF15 and nGen CF 10 specimens, regardless of the raster orientation. In the specimens with the 90°/0° raster orientation, the transversal fibers appeared as white luminous lines on the layers deposited at 0°. In addition, bright spots could be seen on the layers deposited at 90° due to their cross-sectioning. Conversely, in samples manufactured using a −45°/+45° raster orientation, the fibers were primarily visible as luminous points and nonoriented short fibers.

### 3.2. Tensile Tests

**Figure 6:**
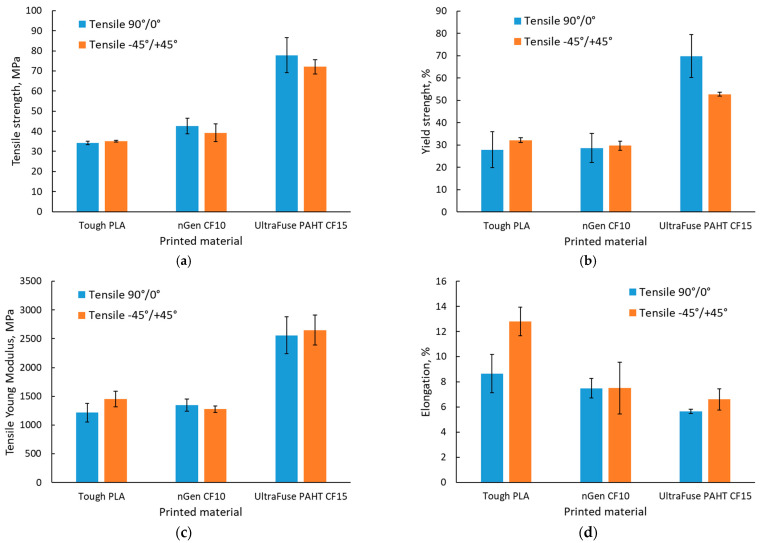
Tensile test results: (**a**) tensile strength; (**b**) yield strength; (**c**) elastic modulus; (**d**) elongation.

Histograms with the average results of the tensile tests for all material batches and raster orientations are presented in Figure 6.

As depicted in the histograms shown in Figure 6, it was observed that the highest tensile and yield strengths were registered for the UltraFuse PAHT CF15 material, as expected due to the higher carbon fiber content, and the lowest tensile strength was registered for the unreinforced PLA. The results obtained for Tough PLA were significantly different from those presented in the material datasheet [36]; in terms of the tensile strength, the average recorded value was 25% lower compared with the value of 46 MPa presented in the material’s datasheet, and in terms of the elastic modulus, the average recorded value was 56% lower compared with the presented value of 2750 MPa. The results obtained for Tough PLA were similar to those obtained for usual PLA studied by other authors [13,14].

Likewise, different values were recorded for nGen CF10 and UltraFuse PAHT CF15 compared with the properties presented in the materials’ datasheets [37,38]. For both materials, the average recorded values were lower compared with the values presented in the materials’ datasheets; for nGen CF10, a 22% lower tensile strength was recorded (compared with 54.71 MPa) and a 54% lower elastic modulus (compared with 2945.78 MPa), while for the UltraFuse PAHT CF15, a 25% lower tensile strength was recorded (compared with 103.2 MPa for dried specimens) and a 70% lower elastic modulus (compared with 8386 MPa).

The values recorded for UltraFuse PAHT CF15, regardless of the raster orientation used, were slightly lower than those reported by Tutar [23] for the framed specimens, and higher than those obtained for the unframed specimens. Despite this, he obtained the highest tensile strength for the framed ±45° manufactured specimens, while in the present case, the 90°/0° raster orientation ensured the highest mechanical performances. These differences could be explained by the different manufacturing conditions used (layer thickness, nozzle and building plate temperature).

**Figure 7:**
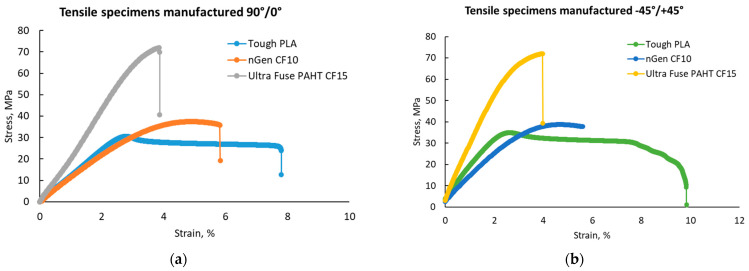
Representative stress–strain curves for the test batches: (**a**) with 90°/0° raster orientation; (**b**) with −45°/+45° raster orientation.

An increase in the tensile strength and elastic modulus simultaneously with a decrease in toughness and ductility with the addition of carbon fibers to polymers was recorded. Moreover, Tough PLA had a more ductile behavior, with higher elongation values regardless of the raster orientation, as can be observed in Figure 7a,b, where a comparison between the stress–strain curves of the materials and raster orientations tested is presented. This behavior was consistent with the findings of other authors [20,21,23].

**Figure 8:**
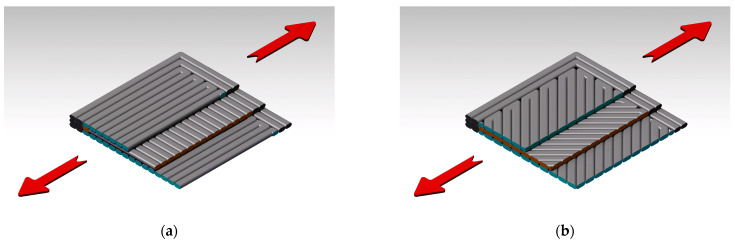
Tensile force direction during the testing of the two configurations: (**a**) 90°/0° raster orientation; (**b**) −45°/+45° raster orientation.

Regarding the influence of the raster orientation, regardless of the material type, a slight influence was observed, especially in the case of the tensile strength and elongation. The 90°/0° raster orientation mainly ensured a higher tensile strength yet the lowest elastic modulus and elongation, while in the case of the −45°/+45° raster orientation, the behavior was opposite, as it mainly ensured the lowest tensile strength yet the highest elastic modulus and elongation. The differences were caused by the direction of the force applied during testing, as can be seen in the representative images in Figure 8. Different colors were used in Figure 8 to highlight the differences between the deposited layers; in Figure 8a, the 90° filaments were marked in cross-section with blue, while the 0° filaments were marked with orange. In Figure 8b, the −45° filaments were marked in cross-section with blue, while the +45° filaments were marked with orange.

**Figure 9:**
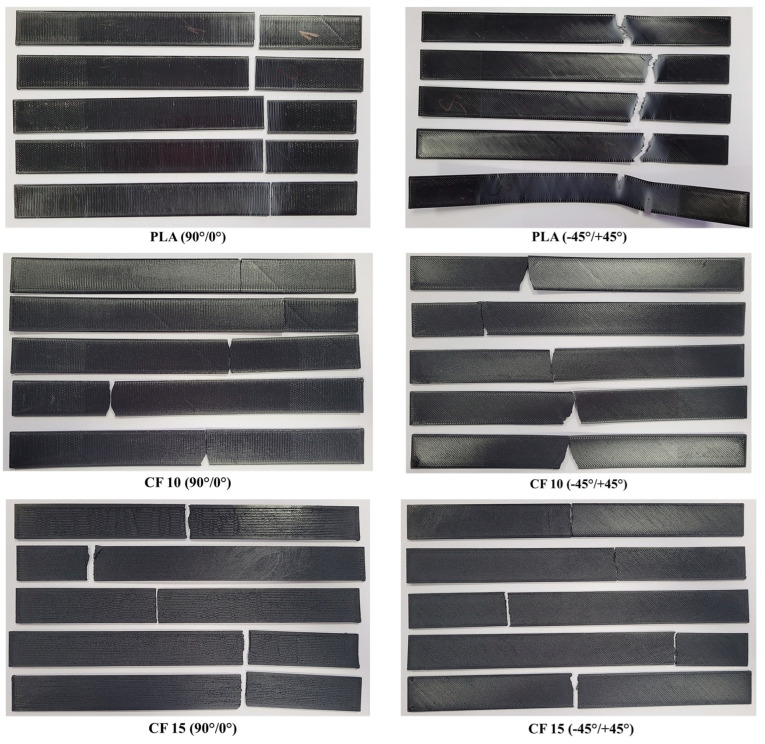
Representative images with all tensile testing specimen batches (**left** side—top of the specimens; **right** side—bottom of the specimens).

A visual inspection determined the specimens’ failure modes, which are presented in Table 4, and images with the fractured specimens are presented in Figure 9. 

Based on the visual specimen analysis, it could be stated that the PLA specimens were the most plastic materials as they elongated more, especially for the −45°/+45° batch. The PLA batch manufactured with a 90°/0° raster orientation showed an LGB main failure mode, while two predominant failure modes were registered for the −45°/+45° batch, namely, AWB and AGB. As the 10% carbon fiber ensured a higher mechanical strength of the nGen CF10 material, it was less ductile than Tough PLA, and many specimens were damaged in the multimode, with the damage starting on the lateral section when the fractured material pieces were detached from the specimen. In the case of UltraFuse PAHT CF 15, clean lateral fractures were identified, and no angled damage was recorded. These fractures were similar to those observed in the case of long-carbon-fiber-reinforced composites.

### 3.3. Fracture Analysis

**Figure 10:**
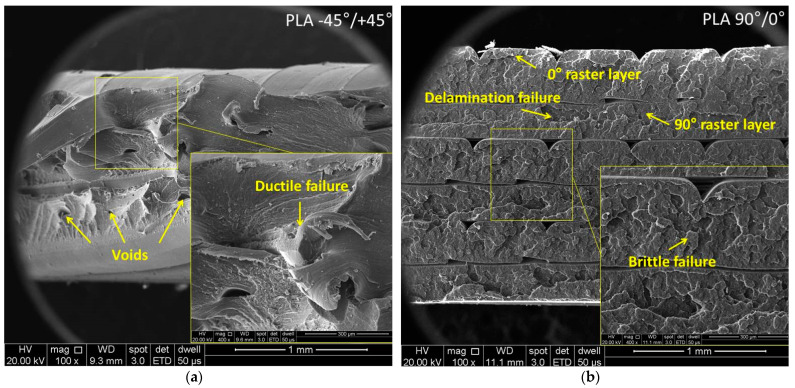

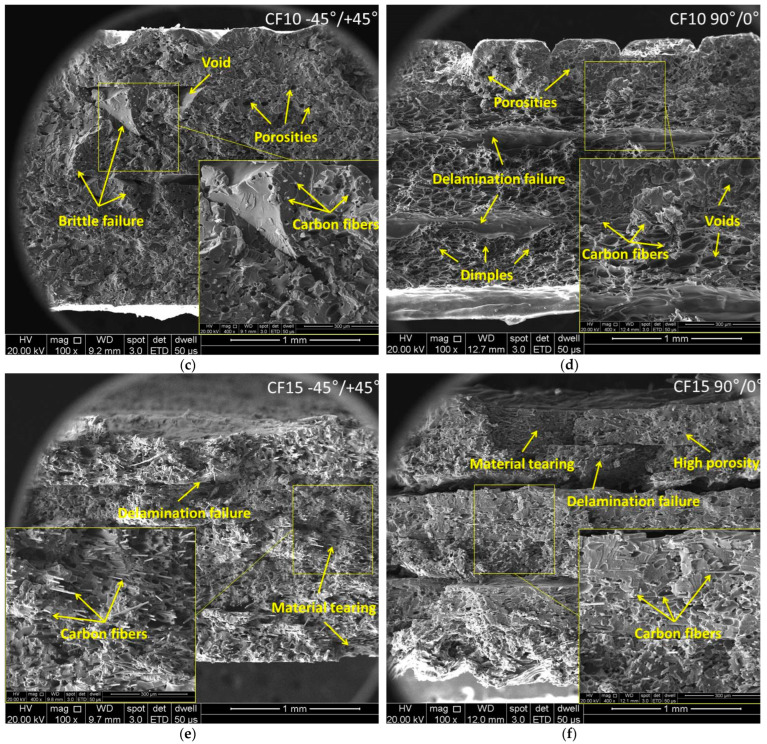
Representative SEM images of fractured surfaces from Tough PLA, nGen CF10 and UltraFuse PAHT CF15 specimens with −45°/+45° and 90°/0° raster orientations: (**a**,**b**) Tough PLA, (**c**,**d**) nGen CF 10 and (**e**,**f**) UltraFuse PAHT CF15.

The goal of the analysis was to locally evaluate the morphology of the fractured areas. Figure 10 shows SEM images with the fractured surfaces of the three materials.

The SEM images of the Tough PLA specimens with a raster orientation of −45°/+45° shown in Figure 10a indicated a predominantly ductile fracture mode and an irregular fracture surface. Despite using the same material and printing conditions, the Tough PLA specimens with a raster orientation of 90°/0° exhibited less ductile behavior compared to the −45°/+45° batch and a uniform fracture surface. This difference in behavior could be attributed to the direction of the applied force during testing, as illustrated in the schematic representation in Figure 8; its impact was also reflected in the variations in tensile properties.

In the case of the 90°/0° batch, triangular-shaped porosities could be noticed between the raster layers, as is shown in Figure 10b. These porosities were caused by the stacking effects inherent to FDM printing that ensure an incomplete material filling among the deposited layers. These porosities have a critical role in determining the interlayer adhesion and, consequently, the mechanical properties of the specimens. The fewer pores, the better the interlayer adhesion, material density and mechanical properties. However, it was noticed that the Tough PLA specimens had higher elongation values compared to the nGen CF 10 and UltraFuse PAHT CF15 specimens. On the other hand, the Tough PLA specimens exhibited a lower tensile strength compared to the nGen CF 10 specimens and especially UltraFuse PAHT CF15 specimens, which demonstrated the highest tensile strength among the three materials. The superior mechanical behavior observed in the UltraFuse PAHT CF15 specimens was primarily related to the higher amount of carbon fibers embedded in the matrix of the specimens. Figure 10c–f illustrate that the carbon fibers integrated in the matrix had a nominal diameter of 8 µm. Even a slight increase of 5 wt.% in the amount of short carbon fibers used in the UltraFuse PAHT CF15 specimens compared to the nGen CF10 specimens resulted in remarkable improvements in the elastic modulus and breaking strength for both raster orientations. The main fracture mechanisms for both materials were fiber breakage and fiber pull-out. Ultimately, the failure of the specimens was attributed to a combination of material tearing and delamination. However, the fracture behavior and tensile properties of both the nGen CF10 and UltraFuse PAHT CF15 specimens exhibited distinct characteristics depending on the chosen raster orientation. The specimens were printed using an alternating distribution of a 0°/90° or −45°/+45 raster angle, leading to different fracture behaviors. In the case of the 90°/0° raster orientation, the tensile load was exerted parallel in the direction of the layers deposited at 90°, and perpendicular on the layers deposited at 0°. This loading configuration influenced the interlayer adhesion, fiber orientation and overall mechanical response of the specimens. Consequently, the failure mechanism depended on the adhesion between adjacent raster layers. It was observed that less tensile load was required to fracture the specimens with a −45°/+45° raster orientation due to the force that was applied at a 45° angle, ensuring a lower tensile strength but a higher elongation.

In the case of the UltraFuse PAHT CF15 specimens, the voids formed during printing acted as sites for crack nucleation, which, ultimately, led to severe delamination between the layers, as shown in Figure 10f. Despite the delamination, these specimens exhibited the highest mechanical strength, particularly when the 90°/0° raster orientation was used. The results indicated that specimens built with the 90°/0° raster orientation demonstrated higher values of tensile strength and elastic modulus while maintaining similar elongation values compared to the −45°/+45° batch.

### 3.4. Open-Hole Tensile Tests

**Figure 11:**
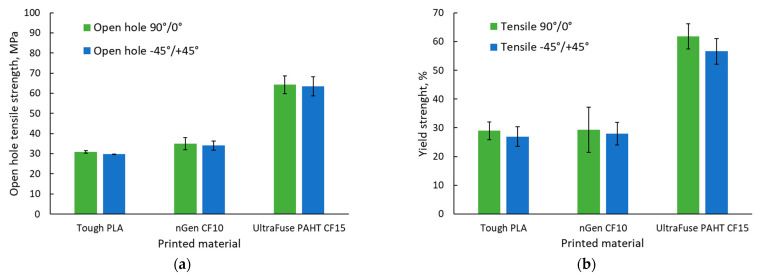

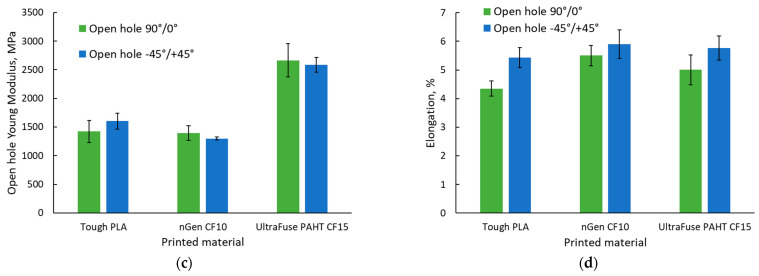
Open-hole tensile test results: (**a**) open-hole tensile strength; (**b**) open-hole yield strength; (**c**) elastic modulus; (**d**) elongation.

The results of the open-hole mechanical tests are presented in Figure 11.

All specimens were damaged according to a standard fracture initiated from the hole area; thereby, no specimen was excluded from analysis. Similar to the tensile tests, the open-hole tensile tests revealed that the highest strength was ensured by the UltraFuse PAHT CF15 material. In this case, we observed that in terms of the strength and elastic modulus, the values recorded were similar regardless of the raster orientation; it seemed that integrating a hole in the middle of the specimen diminished the influence of the raster orientation.

**Figure 12:**
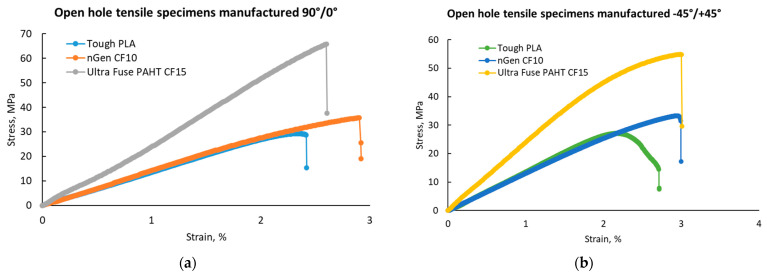
Representative stress-strain curves for the open-hole tensile test batches: (**a**) with 90°/0° raster orientation; (**b**) with −45°/+45° raster orientation.

The −45°/+45° raster orientation provided the highest elongation, according to Figure 11c), as no 0° layers were present. The layers deposited at 0° had the lowest mechanical performance in the testing direction, as they were deposited perpendicular to the applied force direction. Figure 12 presents the representative stress–strain curves for each specimen batch.

The results obtained for the Tough PLA material were similar with the results obtained by Khosravani et al. [43] in the case of usual PLA specimens manufactured with two different hole sizes. They obtained an open-hole tensile strength of 36.9 MPa for specimens with holes of 6 mm and 29.7 MPa for specimens with 12 mm holes.

Comparing the average tensile strength with the average open-hole tensile strength showed that a reduction in the materials’ strength was registered for all materials, although to different extents, as can be observed in Table 5.

**Figure 13:**
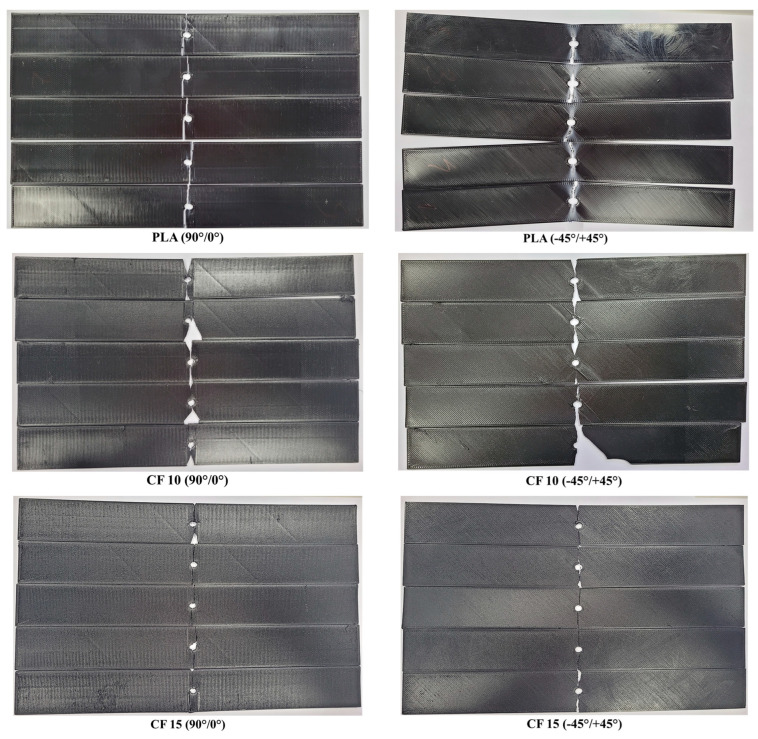
Representative images with all open-hole tensile testing specimen batches (**left**—top of the specimen; **right**—bottom of the specimen).

It was observed that inserting holes in the material reduced the mechanical performance. In the case of a 90°/0° raster orientation, the reduction was more significant in the case of materials with higher mechanical performances, but lower elastic properties (nGen CF10, UltraFuse PAHT CF15). Nevertheless, in the case of the −45°/+45° raster orientation, where the materials’ elastic properties were similar (the elongation values were in a narrower range compared with the case of the other raster orientation), the influence of the hole was more significant for Tough PLA compared with the other two materials. Regarding the damaged specimens, Figure 13 shows the fractured specimens and Table 6 shows their presented failure modes.

Based on the visual specimen analysis, it could be stated that regardless of the raster orientation and material type, the damage originated from the hole area. In the case of materials with higher mechanical strength and toughness, the specimens exhibited multiple modes of failure in various layers, and several pieces of material detached from the specimens from the area adjacent to the hole. The main failure modes were LGM and MGM. The microstructural features and fracture surfaces of the open-hole tensile test specimens did not show any significant differences compared to the monotonic tensile test specimens. Hence, the SEM analysis was not repeated for the open-hole specimens.

### 3.5. Three-Point Bending Tests

**Figure 14:**
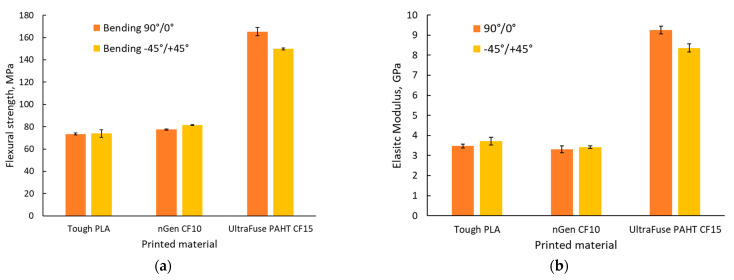

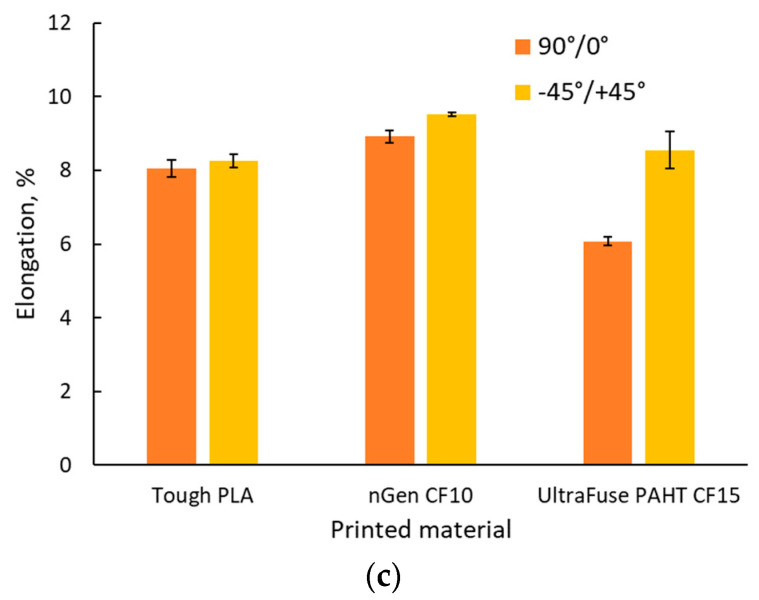
Three-point bending test results: (**a**) flexural strength; (**b**) elastic modulus; (**c**) elongation.

**Figure 15:**
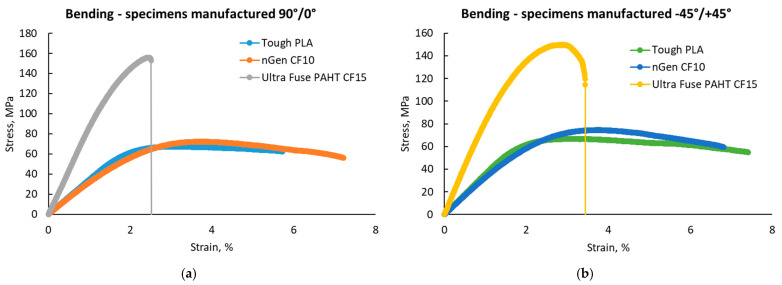
Representative stress–strain curves for the three point bending test batches: (**a**) with 90°/0° raster orientation; (**b**) with −45°/+45° raster orientation.

The flexural test results are presented in Figure 14, while representative stress–strain curves are presented in Figure 15. As was observed in the case of the tensile tests, the UltraFuse PAHT CF15 material had the highest flexural strength and elastic modulus, but the lowest elongation at break. Only a slight difference was recorded between the flexural properties of Tough PLA and nGen CF 10, compared with the significant difference between them and UltraFuse PAHT CF 15. This was caused by the low reinforcement proportion in the case of nGen CF 10, where the short fibers could not ensure a high resistance, but increasing the fiber proportion could also increase the flexural properties.

Studying the materials’ datasheets [36,37,38], we observed that Tough PLA and nGen CF 10 had no information regarding the flexural properties of the materials, but it was provided for UltraFuse PAHT CF15. The results obtained for the flexural properties of UltraFuse PAHT CF15 were similar to those provided in the datasheet for the flexural strength and elastic modulus, regardless of the raster orientation; but, here, we obtained higher elongations at the break.

The results obtained for the flexural strength of Tough PLA were a little lower compared with the values recorded by Atakok et al. [44] in the case of PLA and recycled PLA (Re-PLA). They obtained values in the range of 91–125 MPa for PLA and 89.96–113 MPa for Re-PLA, compared with the average value of 74 MPa obtained during this study. The results obtained for Tough PLA in our case were consistent with the findings of Aveen [45] and Wu [46] in the case of an ultrasonic-consolidated fused-filament-fabricated PLA.

**Figure 16:**
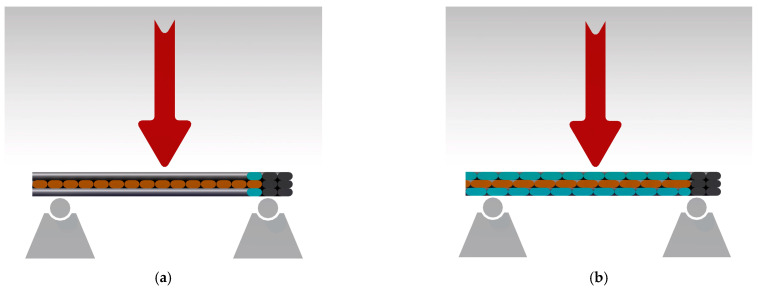
Flexural force direction during the testing of the two configurations: (**a**) 90°/0° raster orientation; (**b**) −45°/+45° raster orientation.

Regarding the applied force direction during flexural testing, the force was applied perpendicularly to the specimens, as shown in the images in Figure 16. The higher flexural strength obtained in the case of the 90°/0° raster orientation was also ensured by the 90° deposited layers (marked with blue in Figure 16a, while orange marks the 0° filament deposition). Figure 16b shows a representation of the −45°/+45° raster orientation cross-section, highlighting the different directions of the filaments in blue for the −45° orientation and in orange for the +45° orientation.

**Figure 17:**
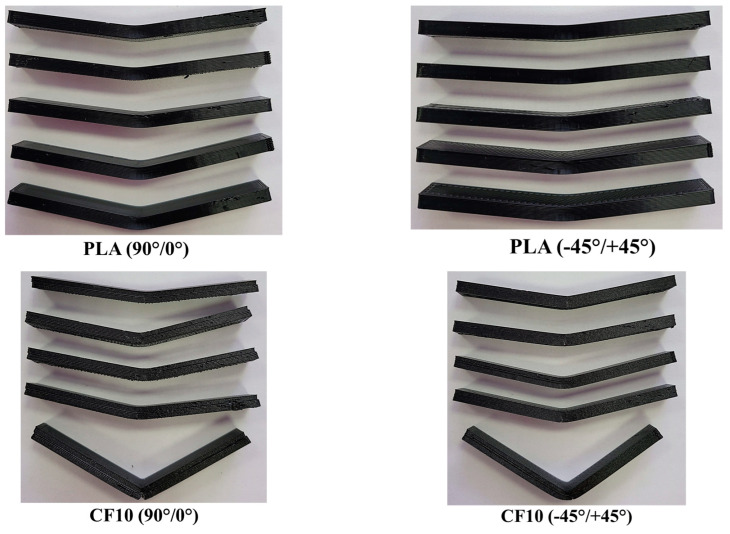

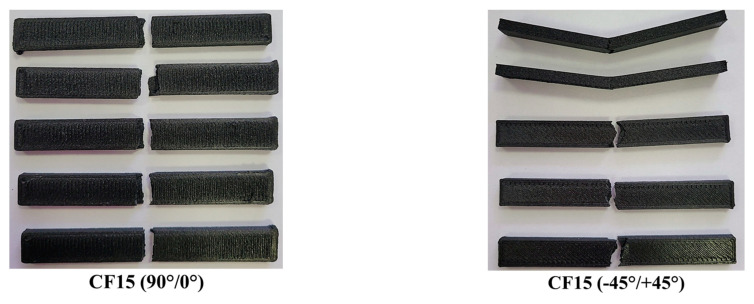
Representative images with all three-point bending specimen batches (**left**—**top** of the specimen; **right**—**bottom** of the specimen).

Figure 17 presents images of the tested specimen batches. The elastic behavior was observed in the case of the Tough PLA specimens, especially for specimens manufactured using a −45°/+45° raster orientation, where they bent during the tests and then returned to their approximate original form afterwards, as can be observed in the images in Figure 17. A complete rupture occurred on the UltraFuse PAHT CF 15 specimens. Similar damage was observed for UltraFuse PAHT CF15 by Tutar [23].

## 4. Conclusions

This study focused on assessing the mechanical properties of three commercially available thermoplastic materials and composites for the FFF process, Tough PLA, nGen CF10 and UltraFuse PAHT CF15, in terms of tensile, open-hole tensile and three point-bending tests. Specimens were manufactured using in-house-developed 3D printing equipment and framed configurations with two different raster orientations, 90°/0° and −45°/+45°.

The results obtained were compared with the existing materials’ datasheets, and it was concluded that some properties were not available in the datasheets and that since manufacturing conditions differed, in some cases, the properties were different as well. Moreover, the open-hole tensile strength of the materials was not a property that was usually found in the materials’ datasheets, even for conventional polymeric composites, but it is important in the case of using the material for perforated parts, as the open-hole represents a tensioned area.

The investigation of the fracture surfaces and tensile properties of the specimens revealed distinct fracture characteristics and varied mechanical properties depending on the chosen raster orientation. The presence of porosities, the amount of carbon fibers and the direction of the applied force all played significant roles in determining the specimens’ behavior under load.

The best mechanical performances were recorded in this case of UltraFuse PAHT CF15. An increase in the tensile, open-hole tensile and flexural strength and elastic modulus simultaneously with a decrease in toughness and ductility was observed in the case of materials reinforced with chopped carbon fibers, regardless of the fiber percentage. The results obtained were mainly similar to the results reported by other authors.

The 90°/0° raster orientations ensured the highest mechanical strengths, regardless of the material type, while the −45°/+45° raster orientations ensured the highest elongation values.

The main conclusion of the study is that before manufacturing parts through the use of AM methods, a material survey should be performed and the materials’ mechanical performances should be studied on many configurations and in different manufacturing conditions.

## Figures and Tables

**Table 1:** Materials’ properties according to their datasheets.

Property	Tough PLA [36]	nGen CF10 [37]	UltraFuse PAHT CF15 [38]	
			Dried Specimen	Conditioned Specimen
Tensile strength (MPa)	46	54.71	103.2 (XY) 18.2 (ZX)	62.9 (XY) 19.1 (ZX)
Tensile stress at break	-	52.26	-	-
Tensile strain at tensile strength	-	3.66	-	-
Yield strength (MPa)	-	54.3	-	-
Yield strain	-	3.75	-	-
Elongation at break (%)	2750	4.56	1.8 (XY) 0.5 (ZX)	2.9 (XY) 0.8 (ZX)
Young’s modulus (MPa)	-	2945.78	8386 (XY) 3532 (ZX)	5052 (XY) 2455 (ZX)
Flexural strength (MPa)	-	-	160.7 (XY) 171.8 (XZ) 50.8 (ZX)	125.1 (XY) 121.9 (XZ) 56.0 (ZX)
Flexural modulus (MPa)	-	-	8258 (XY) 7669 (XZ) 2715 (ZX)	6063(XY) 6260 (XZ) 2190 (ZX)
Flexural strain at break (%)	-	-	2.4 (XY) 2.8 (XZ) 1.8 (ZX)	No break (XY) 3.6 (XZ) 4.0 (ZX)

**Table 2:** Specimens’ dimensions and testing conditions.

	Monotonic Tensile Test	Open-Hole Tensile Tests	Three-Point Bending Tests
Standard	ISO 527-4:1997 [39] and ASTM D 3039/D3039M [40]	ASTM D5766/D5766M [41]	ISO 178 [42]
Dimensions	250 × 25 × 2 mm Span length 150 mm	300 × 36 × 2 mm with a 6 mm hole diameter in the specimens’ centers Span length of 200 mm	80 × 10 × 4 mm Span length 68 mm
Raster orientation	90°/0°		
	−45°/+45°		
Test conditions	Room temperature testing (24°C ± 2°C) Test speed of 5 mm/min. Five specimens/batch		

**Table 3:** Manufacturing conditions.

	Tough PLA	nGen-CF10	UltraFuse PAHT CF15
Nozzle temperature, °C	225	240	260
Building plate temperature, °C	60	85	95
Manufacturing speed, mm/s	40	40	40
Manufacturing plane	XY		

**Table 4:** Failure modes of tensile specimens.

Specimen/ Material	Tough PLA		nGen-CF10		UltraFuse PAHT CF15	
	90°/0°	−45°/+45°	90°/0°	−45°/+45°	90°/0°	−45°/+45°
#1	LGB	AGB	LGB	AGT	LGM	LGM
#2	LGB	AWB	GAB	AAT	GAT	GAB
#3	LWB	AGB	MGB	MGM	LGM	LWT
#4	LGB	AWB	MGT	MGM	LWB	GAB
#5	LGB	LGV	MGM	MGM	LWB	LGM
Main failure type	LGB	AWB/AGB	-	MGM	LWB	GAB

For the first letter: A—angled; L—lateral; G—grip/tab; M—multimode; for the second letter: A—at grip/tab; G—gage; W—<1W from the grip/tab; for the third letter: B—bottom; T—top; M—middle; V—various.

**Table 5:** Reduction in materials’ strength induced by the hole insert.

Raster Orientation/Material	Tough PLA	nGen-CF10	UltraFuse PAHT CF15
90°/0°	9.65%	17.84%	17.35%
−45°/+45°	15.14%	13.01%	11.93%

**Table 6:** Failure modes of open-hole tensile specimens.

Specimen/ Material	Tough PLA		nGen-CF10		UltraFuse PAHT CF15	
	90°/0°	−45°/+45°	90°/0°	−45°/+45°	90°/0°	−45°/+45°
#1	LGM	LGM	MGM	MGM	MGM	MGM
#2	LGM	LGM	MGM	MGM	LGM	MGM
#3	LGM	LGM	MGM	MGM	LGM	LGM
#4	LGM	LGM	MGM	MGM	LGM	LGM
#5	LGM	LGM	MGM	MGM	LGM	MGM
Main failure type	LGM	LGM	MGM	MGM	LGM	MGM

For the first letter: L—lateral; M—multimode; for the second letter: G—gage; for the third letter: M—middle.

## Data Availability

The data presented in this study are available on request from the corresponding author.
